# Advances in Glioblastoma Multiforme Treatment: New Models for Nanoparticle Therapy

**DOI:** 10.3389/fphys.2018.00170

**Published:** 2018-03-19

**Authors:** Elif Ozdemir-Kaynak, Amina A. Qutub, Ozlem Yesil-Celiktas

**Affiliations:** ^1^Department of Bioengineering, Faculty of Engineering, Ege University, Bornova-Izmir, Turkey; ^2^Department of Bioengineering, Rice University, Houston, TX, United States; ^3^Biomaterials Innovation Research Center, Division of Biomedical Engineering, Department of Medicine, Brigham and Women's Hospital, Harvard Medical School, Boston, MA, United States; ^4^Harvard-MIT Division of Health Sciences and Technology, Cambridge, MA, United States

**Keywords:** glioblastoma, delphinidin, nanoparticle, cytoscape, blood-brain barrier modeling

## Abstract

The most lethal form of brain cancer, glioblastoma multiforme, is characterized by rapid growth and invasion facilitated by cell migration and degradation of the extracellular matrix. Despite technological advances in surgery and radio-chemotherapy, glioblastoma remains largely resistant to treatment. New approaches to study glioblastoma and to design optimized therapies are greatly needed. One such approach harnesses computational modeling to support the design and delivery of glioblastoma treatment. In this paper, we critically summarize current glioblastoma therapy, with a focus on emerging nanomedicine and therapies that capitalize on cell-specific signaling in glioblastoma. We follow this summary by discussing computational modeling approaches focused on optimizing these emerging nanotherapeutics for brain cancer. We conclude by illustrating how mathematical analysis can be used to compare the delivery of a high potential anticancer molecule, delphinidin, in both free and nanoparticle loaded forms across the blood-brain barrier for glioblastoma.

## Introduction

Glioblastoma is the most common primary malignant form of brain cancer, with a median survival of 7–15 months from the time of diagnosis. Hallmarks of the aggressive cancer include extensive infiltration and strong vascular proliferation into the surrounding brain parenchyma (Wei et al., 2014; Kim et al., 2015; Van Tellingen et al., 2015). Conventional therapy for glioblastoma, tumor resection followed by radiotherapy and chemotherapy [typically temozolomide (TMZ)], is limited in efficacy due to high rates of recurrence, overall resistance to therapy, and devastating neurological deterioration (Kim et al., 2015; Lin et al., 2015).

Over the past decade, there has been an explosion of research on glioblastoma, with thousands of published reports related to prognosis, treatment response and treatment targets. However, few studies have led to changes in patient outcome (Huang et al., 2013a; Hanada et al., 2014; Thuy et al., 2015). A new approach to brain cancer translational studies is greatly needed to address the overwhelmingly poor treatment results for patients currently diagnosed with glioblastoma. One emerging and promising avenue is nanotechnology based drug delivery (Dilnawaz and Sahoo, 2013; Thuy et al., 2015).

**Figure 1:**
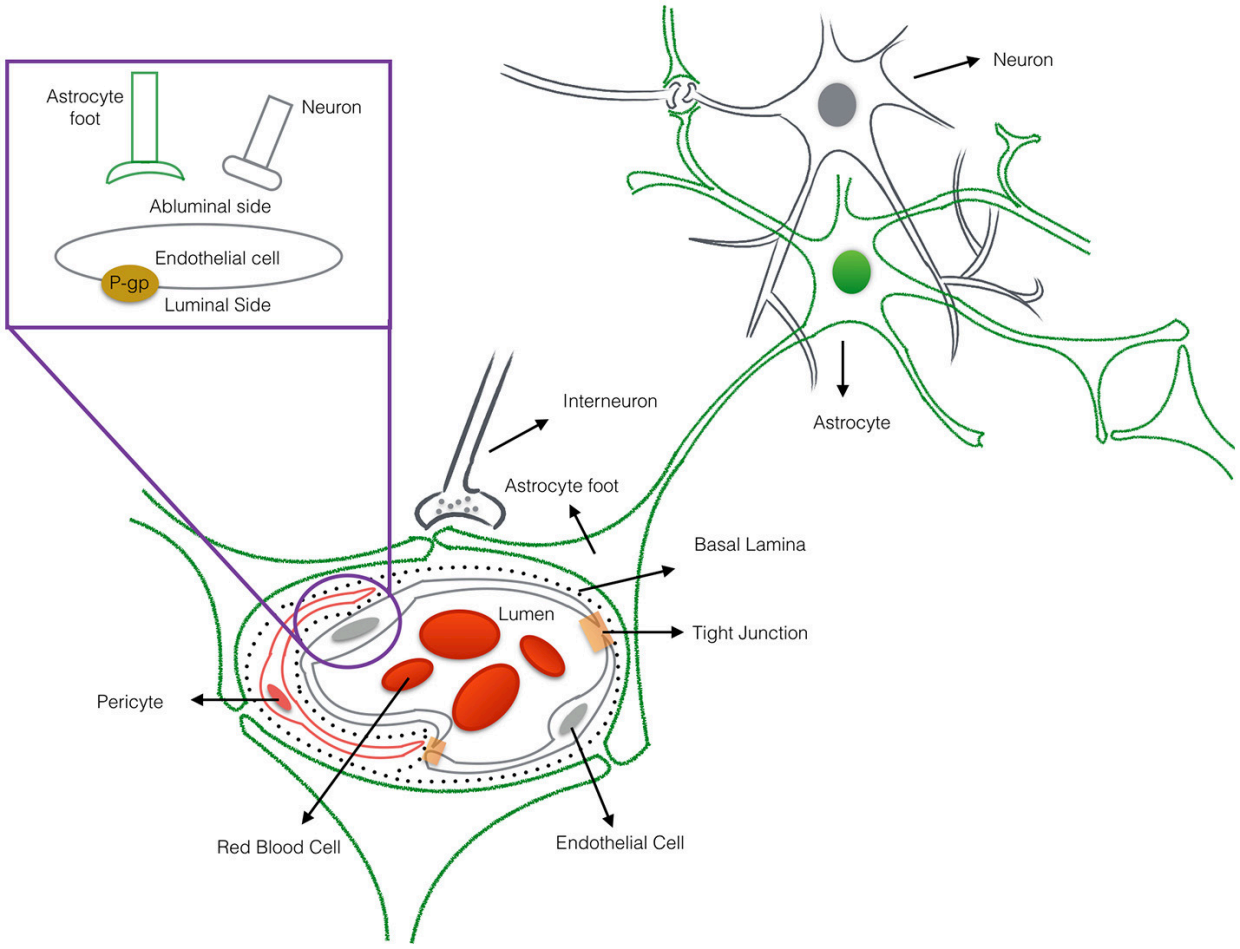
The cross-sectional view of the blood-brain barrier. The blood-brain barrier (BBB) is a physical interface formed by cerebral endothelial cells, separated from pericytes and astrocytic end-feet by the basal lamina. The interactions of the endothelial cells and astrocytes maintaining the integrity of the BBB. Routes for molecular transport across the BBB are not depicted except energy-dependent transport protein (p-glycprotein) which acts as an efflux transporter.

Nanotechnology helps address a major hurdle of glioma therapy: delivery of active compounds to brain tissue. Therapeutics delivery to the brain is limited and controlled by the presence of the blood-brain barrier (BBB), which blocks toxins as well as many essential drugs from reaching brain tissue (Bicker et al., 2014; Kim et al., 2015). The BBB represents a physical interface in the central nervous system (CNS) between the blood and neural tissue (Figure 1). The BBB is comprised of endothelial cells, astrocytes' end-feet, pericytes, and adjacent neurons. The unique interactions of tightly connected endothelial cells and astrocytes maintains the integrity of the BBB, the expression of tight junction proteins, and the prevention of paracellular diffusion (Grover et al., 2014). The BBB also actively functions to exclude substrates from cells through efflux proteins, including ATP-binding cassette transporters and p-glycoprotein (p-gp) (Demeule et al., 2002). The 170 kDa p-gp protein, located at the luminal surface of the brain microvascular endothelium, acts as a “brain gatekeeper” by actively transporting proteins out of the brain capillaries (Figure 1). Those compounds that escape p-gp, must also be lipid soluble small molecules, electro-neutral molecules, or nutrients under 400–600 Da in order to diffuse passively across the BBB's endothelial cell membrane. As such, an estimated 99% of drugs in development fail to cross the BBB, and this severely limits the number of neurological therapies. FDA-approved drugs that effectively cross the barrier are only available for a subset of neurological diseases such as depression, affective disorders, chronic pain, and epilepsy (Aryal et al., 2014; Cheng et al., 2014; Timbie et al., 2015). Advanced delivery methods and disruption of the BBB with nanomedicine techniques are approaches that can help address the bottleneck of brain drug delivery for glioblastoma. However, to capitalize on nanomedicine's benefits, the size, shape, and other nanoparticle delivery properties need to be optimized. To that end, computational models can provide a better understanding of the complex processes involved in delivering effective glioblastoma therapies, and to examine molecular-level interactions in a systemic way to increase therapeutic efficiency (Kim et al., 2013; Matsson and Bergström, 2015).

**Figure 2:**
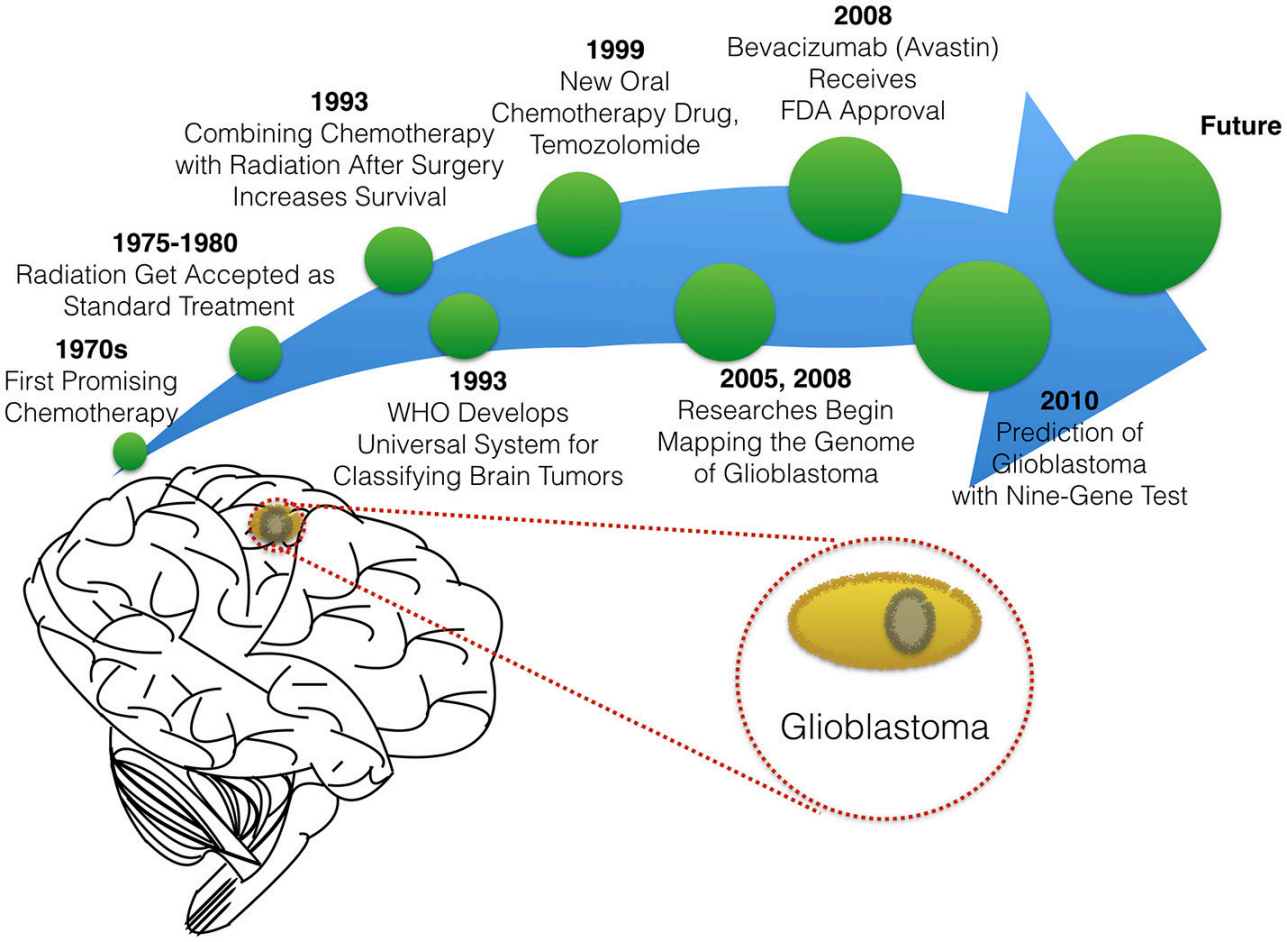
The timeline of glioblastoma therapy.

In this paper, we will briefly summarize milestones that led to the existing therapies in glioblastoma treatment (Figure 2), before reviewing novel experimental and computational approaches to optimizing targeted delivery of active nanoparticles to glioblastoma through the BBB.

## Current therapies for glioblastoma therapy

### Surgery, radiotherapy, anti-angiogenic therapy and precision chemotherapy

The standard treatment for glioblastoma is surgery. This approach includes consideration of maximum surgical resection of tumor tissue even if the entire tumor cannot be removed (Salcman, 1988; Quigley and Maroon, 1991). Even when possible, surgical resection in the case of glioblastoma is limited by the aggressiveness of the glioblastoma which is characterized by infiltration into surrounding tissue and extensive tumor vascularization (Alifieris and Trafalis, 2015; Séhédic et al., 2015). Hence resection surgery is coupled to a course of drug and/or radiation therapy. Chemotherapy for glioblastoma has evolved since the 1970s when researchers first reported data on the efficacy of carmustine (BCNU), a compound which is able to cross the BBB and attack glioma cells directly (Wilson, 1976; Walker et al., 1980). In 1979, Salazar and colleagues proved that radiation therapy is effective against brain cancers (Salazar et al., 1979). Shortly after radiation therapy was developed for treatment in glioblastoma, researchers showed that combining chemotherapy with radiation therapy helps patients live longer (Fine et al., 1993). Today, standard therapy for patients with glioblastoma is a combination treatment, including radiotherapy alone or with chemotherapy, both before and after surgery. Chemotherapy may also be used to delay the need for radiation in younger children.

The choice of drug therapy for glioblastoma is still limited to a handful of compounds. In 1999, Temozolomide, the oral chemotherapy drug was granted accelerated approval by the FDA to treat anaplastic astrocytoma (a form of high-grade glioma) (Yung et al., 1999; Brada et al., 2001). Currently, Temzolomide is the preferred FDA-approved chemotherapeutic agent for glioblastoma (Alifieris and Trafalis, 2015; Frosina, 2015). One feature enabling its success is that Temozolomide treatment can be tailored based on patient characteristics. Researchers discovered that patients with promoter MGMT gene methylation have increased median survival times when given Temozolomide with radiotherapy therapy vs. radiotherapy alone. The MGMT gene enables DNA repair, even where damage was caused by chemotherapy (Hegi et al., 2005; Alifieris and Trafalis, 2015).

The use of Temozolomide therapy for patients with MGMT gene methylation highlights the importance of another growth area in glioblastoma clinical research: increasingly precise classification of tumors and patients. While drugs have been in development, so too have methods to classify and grade brain tumors. The World Health Organization (WHO) developed international standards for classifying brain and nervous system tumors in the 1990s (The new WHO Classification of Tumors affecting the Central Nervous System, 1993). Experts continue to update tumor classifications according to the cell type and tumor malignancy grade as scientific knowledge grows and new glioma subtypes are identified. These classification standards allow doctors and researchers to have a common language for describing and sharing knowledge about tumor stage, characterization, genetics, and treatment.

To help improve predictions of response to therapy, many researchers have searched for genetic cues in glioblastoma patients. The National Cancer Institute and The National Genome Research Institute launched The Cancer Genome Atlas Project in 2005, with the aim of mapping the genetic changes in glioblastoma and other cancers. In 2008, research studies reported the identification of several key mutations that are involved in inducing the development and invasiveness of glioblastoma (TCGA, 2008). In 2010, a set of nine genes were identified to predict the likelihood that a glioblastoma tumor would respond to therapy. The multivariate diagnostic test called DecisionDx-GBM, was created to determine the molecular signature of glioblastoma tumors and help identify the most effective existing therapy and/or suggest new treatments targeting tumors that do not respond to the standard therapies (Colman et al., 2010).

**Figure 3:**
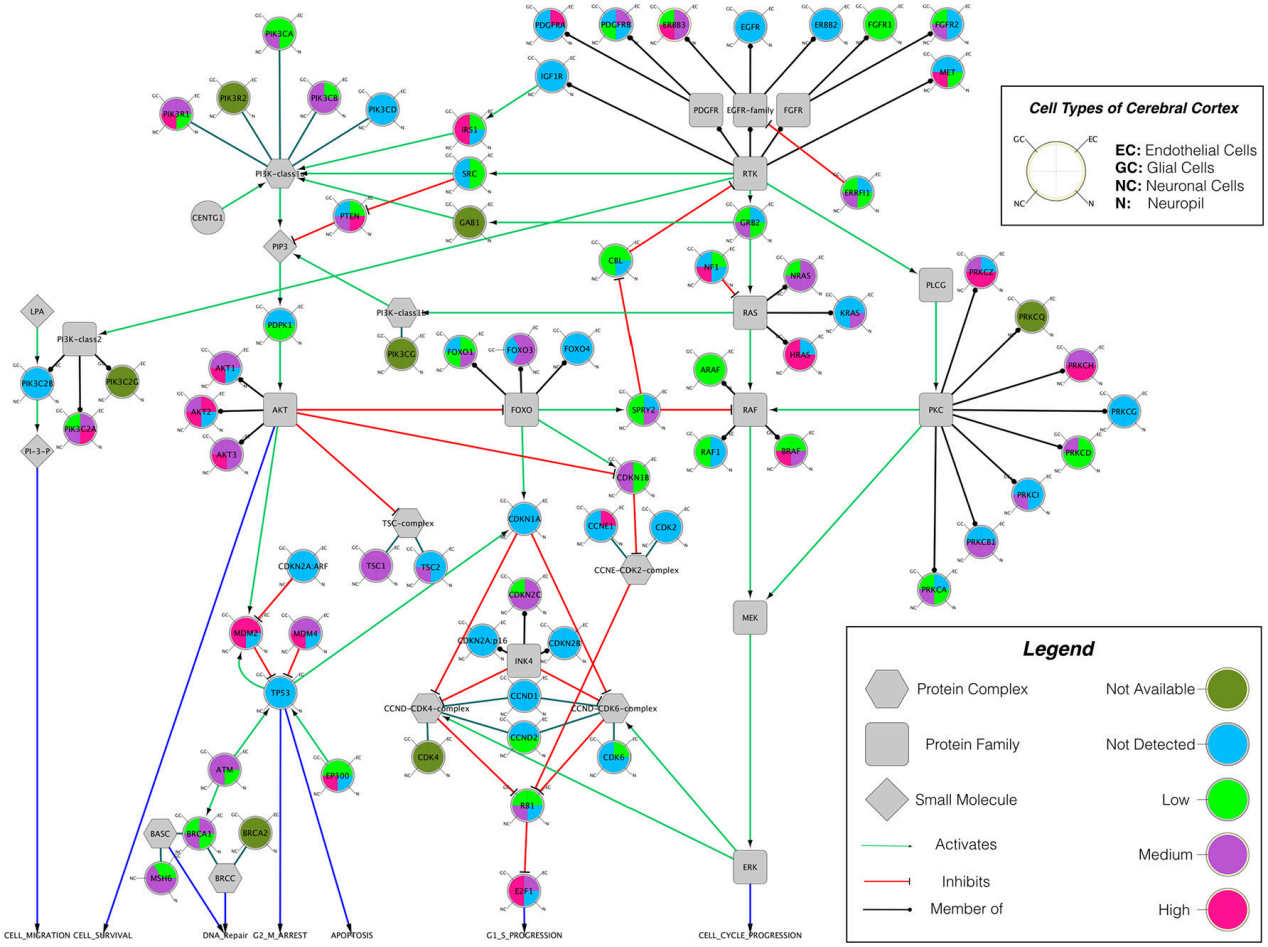
The protein expression levels of healthy cerebral cortex cells mapped onto the GBM pathway map from the TCGA data set. In this diagram, The Human Protein Atlas database was used to obtain protein expression levels of healthy cerebral cortex cells. Special shapes used in the map represent different types of molecules which are given in the legend of the figure as protein complex, protein family, protein or small molecule. Lines and arrows show the relationship of the molecules. Each protein symbol (circle) is divided into quarters to represent, in a clockwise order: endothelial cells, neuropil, neuronal cells, and glial cells. The corresponding protein expression levels are shown in different colors. Olive for not available, deep sky blue for not detected, green for low expression, medium orchid for medium expression, and deep pink for high expression. The original GBM pathway map in the Cytoscape format was downloaded from (“The Cancer Genomics at cBio—Glioblastoma (TCGA)” n.d.).

**Figure 4:**
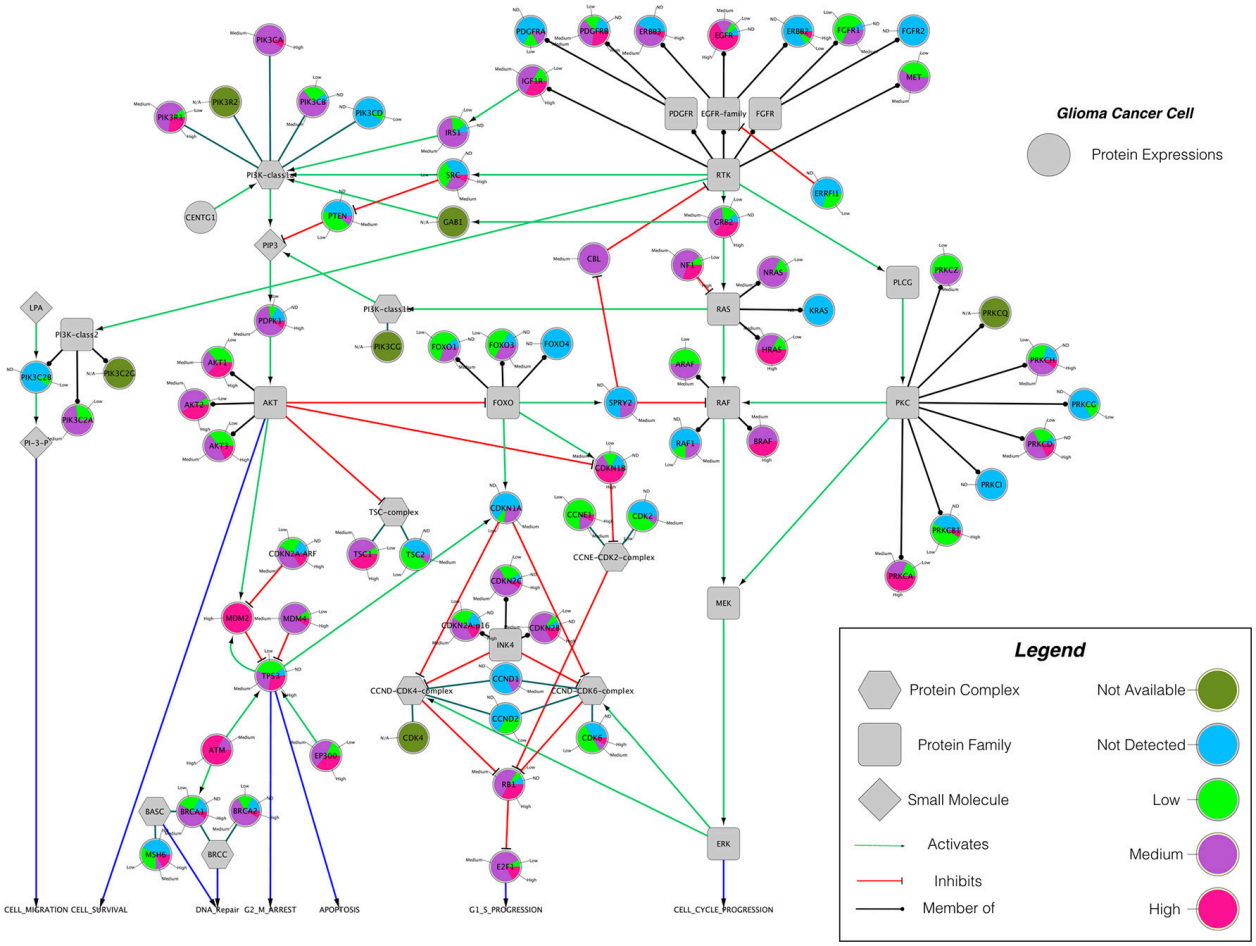
The protein expression levels of glioma cancer cells mapped onto the GBM pathway map from the TCGA data set. In this diagram, The Human Protein Atlas database were used to obtain protein expression levels of glioma cancer cells. Special shapes used in the map represent different types of molecules which are given in the legend of the figure as protein complex, protein family, protein and small molecule, respectively. Lines and arrows show the relationship of the molecules. Each protein symbol (circle) is shown as pie chart to represent different expression levels of glioma cancer cell which are depicted in different colors. Olive for not available, deep sky blue for not detected, green for low expression, medium orchid for medium expression, and deep pink for high expression. The original GBM pathway map in the Cytoscape format was downloaded from (“The Cancer Genomics at cBio—Glioblastoma (TCGA)” n.d.).

Building on important progress in understanding the molecular pathogenesis of malignant glioblastoma (McLendon et al., 2008; Wen and Kesari, 2008), we summarize known gene interactions and protein expression patterns in glioblastoma through a signaling network diagram representation (Figures 3, 4). Specifically, we analyzed aberrations in signaling pathways for glioblastoma cells compared to four different types of healthy cerebral cortex cells. Glioblastoma signaling pathway map served as our default template (“The Cancer Genomics at cBio—Glioblastoma (TCGA)”^1^ n.d.) and networks of related differentially expressed genes were rendered using Cytoscape. We extended and supplemented the original TCGA pathway map using protein expression levels of tumor cell and healthy cerebral cortex cells obtained from The Human Protein Atlas (Table 1). Each protein symbol is divided into quarters to represent, in a counterclockwise order: (1) endothelial cells, (2) glial cells, (3) neuronal cells, and (4) neuropil of the healthy cerebral cortex; the corresponding protein expression levels are shown in different colors (Figure 3). The analogous protein expression levels for glioma cancer cells are depicted as well (Figure 4). Proteins that are differentially expressed in glioma compared to the healthy cortex are highlighted in the context of known molecular signaling pathways. Recent studies have shown promising results of targeting one of the displayed over-expressed receptors using a nanodelivery system to the glioblastoma tumor (Krakstad and Chekenya, 2010; Qin et al., 2014; Weber and Ryan, 2015; Whittle et al., 2015). Future drug development can capitalize on these and similar analyses of known signaling mechanisms in glioma in order to help develop cell- and patient-specific targeted therapies.

**Table 1:** Protein classes, names and expression levels in the cerebral cortex and brain tumor which is obtained from www.proteinatlas.org.

**Class**	**Protein**	**Cerebral cortex**				**Cancer**			
		**Endothelial cells**	**Glial cells**	**Neuronal cells**	**Neuropil**	**Not detected**	**Low**	**Medium**	**High**
PI3K (Class 1a)	PIK3CB	Low	Medium	Medium	Medium	1/11	3/11	7/11	0
	PIK3R2	N/A				N/A			
	PIK3CA	Low	Low	Medium	Low	0	0	10/11	1/11
	PIK3R1	Medium	Medium	High	Low	0	1/11	7/11	3/11
	PIK3CD	Not Detected				11/12	1/12	0	0
PI3K (Class 1b)	PIK3CG	N/A				N/A			
PI3K (Class 2)	PIK3C2B	Not Detected				9/10	1/10	0	0
	PIK3C2A	Medium	Low	Medium	High	0	3/11	8/11	0
	PIK3C2G	N/A				N/A			
TSC Complex	TSC1	Medium	Medium	Medium	Medium	0	1/12	6/12	5/12
	TSC2	Not Detected	Not Detected	Medium	Not Detected	4/10	5/10	1/10	0
BASC	MSH6	?	Low	Medium	Medium	5/12	4/12	1/12	2/12
BASC/BRCC	BRCA1	Medium	Low	Medium	Low	2/12	3/12	6/12	1/12
BRCC	BRCA2	N/A (RNA-based expert annotation could not be performed.)				2/12	2/12	7/12	1/12
CCNE-CDK2	CCNE1	High	Not Detected	Not Detected	Not Detected	0	9/12	2/12	1/12
	CDK2	Not Detected				5/11	5/11	1/11	0
CCND-CDK4	CDK4	N/A				N/A			
CCND-CDK6	CDK6	Low	Not Detected	Not Detected	Not Detected	4/12	6/12	1/12	1/12
AKT	AKT1	Medium	Medium	High	Not Detected	0	4/11	3/11	4/11
	AKT2	High	Medium	High	Not Detected	0	1/12	6/12	5/12
	AKT3	Medium	Medium	High	Medium	0	4/11	5/11	2/11
FOXO	FOXO1	Low	Not Detected	Low	Medium	1/10	6/10	3/10	0
	FOXO3	Medium	Not Detected	Medium	?	2/12	6/12	4/12	0
	FOXO4	Not Detected				12/12	0	0	0
PDGFR	PDGFRA	High	Not Detected	Not Detected	Not Detected	8/12	2/12	2/12	0
	PDGFRB	Medium	Not Detected	Low	Not Detected	2/11	2/11	4/11	3/11
EGFR	ERBB3	Medium	Low	High	Medium	5/12	0	6/12	1/12
	EGFR	Not Detected				1/12	1/12	2/12	8/12
	ERBB2	Not Detected				10/12	1/12	0	1/12
FGFR	FGFR1	Low	Low	Low	Low	1/12	7/12	4/12	0
	FGFR2	Not Detected	Low	Medium	Not Detected	12/12	0	0	0
RTK	IGF1R	Not Detected				0	2/12	6/12	4/12
	MET	Not Detected	Not Detected	High	Low	0	5/12	7/12	0
RAS	NRAS	Medium	Low	Medium	Medium	0	2/12	10/12	0
	KRAS	Not Detected	Not Detected	Not Detected	Medium	12/12	0	0	0
	HRAS	Not Detected	High	High	High	0	2/12	4/12	6/12
RAF	ARAF	Low	Low	Low	Low	0	6/12	6/12	0
	BRAF	Low	Low	High	Medium	0	0	6/11	5/11
	RAF1	Not Detected	Low	Low	Not Detected	7/12	2/12	3/12	0
PKC	PRKCZ	Not Detected	Medium	High	Medium	0	6/11	5/11	0
	PRKCQ	N/A				N/A			
	PRKCH	Medium	Medium	High	High	2/10	3/10	4/10	1/10
	PRKCG	Not Detected				10/12	2/12	0	0
	PRKCD	Low	Medium	Low	Low	1/12	3/12	6/12	2/12
	PRKCI	Not Detected	Not Detected	Medium	Not Detected	12/12	0	0	0
	PRKCB	Not Detected	Not Detected	Medium	Medium	5/12	6/12	0	1/12
	PRKCA	Not Detected	Low	Medium	Low	0	2/11	3/11	6/11
INK4	P16(CDKN2A)	Not Detected				2/12	3/12	5/12	2/12
	CDKN2B	Not Detected				1/12	1/12	8/12	2/12
	CDKN2C	Medium	Low	Medium	Medium	1/12	3/12	7/12	1/12
Protein	PDPK1	Not Detected	Not Detected	Low	Low	2/11	1/11	7/11	1/11
	PTEN	Low	Not Detected	Medium	High	4/9	4/9	1/9	0
	IRS1	Low	High	High	Not Detected	1/11	2/11	8/11	0
	SRC	Low	Not Detected	Not Detected	Low	4/12	4/12	3/12	1/12
	GAB1	N/A				N/A			
	ERRFI1	Not Detected	Low	Medium	Low	7/10	3/10	0	0
	GRB2	Not Detected	Low	Medium	Low	1/11	2/11	4/11	4/11
	NF1	Low	Not Detected	High	Not Detected	0	1/10	6/10	3/10
	CBL	Low	Low	Low	Not Detected	0	0	9/9	0
	SPRY2	Not Detected	Low	Medium	Medium	9/12	0	3/12	0
	CDKN1A	Not Detected				7/11	1/11	3/11	0
	CDKN1B	Low	Medium	Medium	Low	2/12	2/12	2/12	6/12
	CCND1	Not Detected				10/12	0	2/12	0
	CCND2	Not Detected	Not Detected	Low	Low	8/12	4/12	0	0
	RB1	Low	Low	Medium	Not Detected	1/12	1/12	6/12	4/12
	E2F1	Medium	High	High	Not Detected	0	1/12	9/12	2/12
	ARF(CDKN2A)	Not Detected				2/12	3/12	5/12	2/12
	MDM2	High	High	High	Not Detected	0	0	0	12/12
	MDM4	Medium	Medium	High	Low	0	1/11	9/11	1/11
	TP53	Not Detected				1/11	4/11	3/11	3/11
	EP300	Low	Low	High	Not Detected	0	2/11	5/11	4/11
	ATM	Medium	Medium	Medium	Low	0	0	2/12	10/12
Small molecule	LPA	Not Detected				4/12	7/12	1/12	0

Ongoing research into angiogenesis also has offered hopeful glioblastoma targets based on the hypothesis that cutting off a tumor's blood supply could starve growing cancer cells. Bevacizumab (Avastin), a drug targeting vascular endothelial growth factor (VEGF-A), was suggested as an early-stage trial of targeted therapy for brain cancer. Avastin is an anti-angiogenic drug that interferes with the development of blood vessels essential to tumor growth and invasiveness (Friedman et al., 2009). The FDA granted accelerated approval for Bevacizumab, based on its efficacy in treating recurrent glioblastoma (Whittle et al., 2015). However, Bevacizumab's use has been rife with controversy. Anti-angiogenic therapy has failed to show improvement for patient overall survival, while still showing efficacy in shrinking or halting tumor growth (Francescone et al., 2012). As a result of lackluster responses by glioblastoma patients to diverse chemotherapies and anti-angiogenic compounds, radiation-combined therapies are considered essential and unavoidable. However, a drawback of radiation is its severe side effects, includes DNA lesions, cognitive impairment, and other systemic effects (Séhédic et al., 2015).

Given the limitation of all current therapeutics (whether surgery, chemotherapy and/or radiation), development of novel approaches to treating glioblastoma remains a great need. To be effective, any new therapy should be specific and controllable and should be able to cross the BBB; moreover it needs to show efficacy. A suite of new nanomedicines have emerged to fill this niche. To that end, we focus the remainder of this review on emerging nanomedicines for glioblastoma and the methods used to study them.

### Nanomedicine

First a definition of what constitutes nanomedicine: Living organisms hold innate nanoscale functional components including proteins which have an average size of about 5 nm, and DNA molecules, which are on the order of 2.5 nm in diameter (Salata, 2004; Kawadkar et al., 2011). Nanotechnology methods have emerged to help understand the biological processes that occur at this nanoscale level. These tools form the basis of the nano-biotechnology field, which integrates biology, physics, and chemistry (Kawadkar et al., 2011) Application of nano-biotechnology to medicine is called nanomedicine, a subfield which has contributed to new directions in drug development, discovery, and delivery for treating malignant brain tumors (Nduom et al., 2012; Zhou et al., 2012; Aryal et al., 2014; Fakhoury, 2015; Jo et al., 2015; Lauzon et al., 2015; Fernandez-Piñeiro et al., 2017). Drug administration to or within tissues of the CNS faces significant challenges including toxicity and BBB crossing. These challenges are compounded in brain cancer by the complex tumor microenvironment, invasive tumor cells, and cancer-associated changes in metabolism (Wei et al., 2014; Séhédic et al., 2015). Nanomedicine offers a potential means to optimize delivery of drugs to brain tumors, by enabling better permeability through the BBB and specific targeting of tumor cell subtypes and of processes in the tumor microenvironment (e.g., tumor stem cells, acidosis) (Jain and Stylianopoulos, 2010; Huang et al., 2013b; Hanada et al., 2014; Wei et al., 2014). Nanoparticle-mediated delivery systems can extend the life-span of active drug compounds and provide for their controlled, continued and local release within brain tissue. To enable their efficacy, it is critical to regulate the physiochemical properties of the delivery carriers, such as their size, overall surface charge, and chemical composition. Careful analysis to determine a nanoparticle's cytotoxicity, biocompatibility, and biodegradability are all needed prior to clinical application to the brain (Jo et al., 2015; Lauzon et al., 2015). A detailed, quantitative understanding of how physicochemical properties of nanoparticles affect therapeutic delivery and efficacy is necessary to optimize the design of nanoparticles for the treatment of brain diseases. Computational modeling can address this need.

### Computational models

Many computational models have been developed to represent some aspects of glioblastoma, and the developed simulation tools can be utilized to predict tumor expansion and understand the unique tumor microenvironment (Gevertz et al., 2008). Overall, models can be classified into different categories varying from minimalistic models simulating just the growth of the tumor volume to molecular-detailed models including many genetic or proteomic processes involved in the development and progression of glioblastoma (Watanabe et al., 2016). As researchers become more aware of the complexity of the biology, modeling approaches have evolved to provide insights into glioblastoma across multiple length scales (tissue, cellular, and molecular) (Gevertz, 2011; Watanabe et al., 2016). While there is an extreme amount of diversity in the glioblastoma models' details and scope, the common aim in all models is to reliably predict certain features of tumor progression to regulate, prevent, or reverse the invasive glioma growth pattern (Gevertz et al., 2008). The more accurately this tumor growth is predicted, the more reliably therapy can be optimized for each cancer patient. Overall models have focused on three main glioma behaviors: vascularization, diffusion, and invasion capacity (Eikenberry et al., 2009; Tektonidis et al., 2011; Alfonso et al., 2016). The models consider key parameters like hypoxia which correlates strongly with glioma invasiveness and malignancy; immune response dynamics which can result in tumor regression and provide therapeutic benefits; and diffusion of therapeutic nano-particles in a three-dimensional space representing the brain's geometry and heterogeneity (Eikenberry et al., 2009; Böttger et al., 2012; Schlüter et al., 2012; Alfonso et al., 2016; Reppas et al., 2016; Watanabe et al., 2016; Rutter et al., 2017).

Computational modeling has the ability to provide predictive and explanatory frameworks for glioblastoma nanoparticle design and delivery, that *in vitro* and *in vivo* glioblastoma models lack (Bicker et al., 2014). Specifically, computational modeling can be used to study the characteristic high heterogeneity of the tumor microenvironment, and predict effects of disrupting molecular pathways in specific brain regions (Escribá et al., 2015). Molecular signaling pathway analysis like that described in the previous section can help quantitatively highlight potential cancer targets (Figures 3, 4), while mathematical models of mass transport phenomena enable predictions of drug delivery to the brain, and they can help in the design of experiments (Lauzon et al., 2015). A variety of exciting mathematical models have been developed to study and predict the progression of glioblastoma (Frieboes et al., 2013; Branco et al., 2014; Martirosyan et al., 2015), including patient-specific ones (Rockne et al., 2004; Swanson et al., 2011; Neal et al., 2013; Colombo et al., 2015).

In parallel with the increase in models of glioblastoma progression, there have been a growing number of studies focused on quantitative approaches to study and improve nanoparticle delivery to the CNS across the BBB (Huang et al., 2009). Effect of nanoparticle formulation, shape, and binding properties on delivery across the BBB has been studied previously both theoretically and in biological studies (Gosk et al., 2004; Takae et al., 2005; Chithrani et al., 2006; Decuzzi and Ferrari, 2008; Fakhari et al., 2011). There are also a few studies which show alternative access into the CNS across the epithelial blood-cerebrospinal fluid barrier (Langlet et al., 2013; Langlet, 2014). However, while diffusion-kinetic models for drug release into brain tissue have been developed for years (Saltzman and Radomsky, 1991; Bandara et al., 2007; Groh et al., 2014), no models have yet explored the optimization of novel anthocyanidin-based compounds and their CNS delivery for treatment of glioblastoma.

To address this knowledge gap, we focus the following sections on the delivery of delphinidin, an anthocyanidin and antioxidant, as a means to both (1) highlight an emerging new nanomedicine in development for glioblastoma, and (2) illustrate how mathematical analysis can be used to improve the design of delphinidin and related new compounds. A summary of presented modeling studies has been show in Table 2.

**Table 2:** The summary of glioblastoma modeling and delivery systems modeling (ECM, Extra cellular matrix; Coeff., Coefficient; PDE, Partial differential equations; GBM, Glioblastoma; ODE, Ordinary differential equations).

**Glioblastoma modeling**	**Model methodology**		**Procedure**	**References**
	Discrete	Three Phase Model	ECM: third phase	Gevertz et al., 2008
			Diffusion coeff. reduced	
			Valid representation of brain microstructure	
			Focused on how microstructural changes impact the transport of nutrients and signaling molecules in the brain	
	Discrete	Lattice Gas Cellular Automaton Model	Cell migration and cell kinetics	Böttger et al., 2012
			Allow for parallel synchronous movement	
			Fast updating of a large number of cells	
			Well-suited for modeling tumor growth and invasion	
	Continuous	The Glioma-Vasculature Interplay Model	The growth of vascularised gliomas	Alfonso et al., 2016
			Focused on the interplay between the migration/proliferation dichotomy and vaso-occlusion at the margin of viable tumor tissue	
			Formulated as a system of reaction-difusion PDEs	
			Go or Grow mechanism	
	Continuous	Reaction-Diffusion Model	Stochastic PDEs that can predict the likely behavior of a given GBM	Eikenberry et al., 2009
			Estimates spatial probability distribution of tumor recurrence	
			Applicable technique to clinical cases of GBM	
	Continuous	Functional Collective Cell-Migration Units (FCCMU) Model	Describes the large-scale morphology and 3-D cell spatial arrangements during tumor growth and invasion and incorporate micro-macro functional relationships	Frieboes et al., 2007
			Based on mass and momentum conservation laws	
			Conserved variables that describe the known determinants of glioma (e.g., cell density)	
			Parameters that characterize a specific glioma tissue	
	Hybrid	2D-Cellular Automaton Model	Explores the feedback that occurs between a growing tumor and the evolving host blood supply	Gevertz, 2011
			Tested using both an angiogenesis inhibitor and a vascular disrupting agent	
	Hybrid	Agent Based Model	3D- multiscale agent based tumor model	Zhang et al., 2007
			Simulates gene-protein interaction profiles, cell phenotypes and multicellular patterns in brain cancer	
	Non-stochastic	One Dimensional Model	Time evolution of the tumor volume before and after a radiosurgical procedure	Watanabe et al., 2016
			The tumor growth rate decreases as the tumor volume increases	
			Some radiation-damaged cells still keep dividing for a few more cell cycles after a single pulse of irradiation	
**Delivery systems modeling**	**Model**		**Procedure**	**References**
	Nanoparticle Mediated	Growth Factor Delivery	Growth factor delivery from the nasal cavities and blood capillaries to the brain tissue holds many modeling challenges	Lauzon et al., 2015
			The main mass transport phenomena involved in NPs as well as GFs inside them	
			How they can be described mechanistically	
	Penetration into tumor tissues	Anti-cancer Drug	Predicts spatio-temporal distributions of drugs within the tumor tissue	Kim et al., 2013
			Simulates different ways to overcome barriers to drug transport	
			Optimizes treatment schedules	
	Quantitive Model Based on ODEs	Paclitaxel	Describes this process of exclusion	Bandara et al., 2007
			Comprises diffusion across both the luminal and the abluminal membrane of brain capillaries	
			Binding in the lumen and in the endothelial cells	
			Active transport of free drug by p-gp from the endothelial cells to the lumen	
	Ligant-Based and Structure-Based	Transport protein on drug influx and efflux	Understanding the substrate and inhibitor interactions with these membrane-bound proteins are discussed	Matsson and Bergström, 2015
			Molecular-level interactions have been developed for a number of important transporters	
	Direct and Nanoparticle Encapsulated Delivery	Delphinidin	Predicts delivery of delphinidin by itself	Our model
			Predicts delivery of nanoparticle-encapsulated forms of delphinidin to brain tissue	
			Estimates mass transport relationships	
			First order, chemical-kinetic, ODE models	
			Convection-diffusion equations	
			Molecular signaling pathway analysis can be used to help develop cell- and patient-specific targeted therapies	

## Optimizing nanomedicine: transport of delphinidin across the blood brain barrier

Derivatives of the anthocyanin family are nanoparticles of great recent interest for treating multiple diseases (Amin et al., 2017; Kim et al., 2017). Antioxidant properties of anthocyanin active molecules, such as delphinidin synthesized from berries, provide potency across diseases: cited health benefits include reduced risk of coronary heart disease, reduced risk of stroke, anti-tumor properties, anti-inflammatory effects, and improved cognitive behavior (Martins et al., 2011; Chakrabarti and Ray, 2015). Notable for applications to glioma, studies have shown anthocyanins selectively inhibit the growth of human tumor cells while enabling normal growth of healthy cells (Galvano et al., 2004). Furthermore, delphinidin can cross the BBB and is taken up by brain tissue, in a process mediated by p-gp efflux (Andres-Lacueva et al., 2005; Chakrabarti and Ray, 2015).

To systematically test the effects of different anthocyanin derivatives on glioblastoma progression, we developed a computational model for the delivery of direct and encapsulated forms of delphinidin to glioblastoma through the BBB. Specifically, we developed two models: (1) to predict delivery of delphinidin by itself and (2) to predict delivery of nanoparticle-encapsulated forms of delphinidin to brain tissue. In both cases, we can estimate mass transport relationships. In the first model, we need four aggregated kinetic terms (diffusivity, binding, dissociation, and active transport) that enable us to define the following processes: (1) passive diffusion across the luminal and the abluminal membrane of brain capillaries, (2) active transport of delphinidin from the endothelial cells to the vascular lumen by p-gp, and (3) release of the compound by control of p-gp activity. In the nanoparticle delivery model, we instead include (1) transport of the nanoparticles from the blood stream through the BBB into the brain parenchyma; and (2) release of the delphinidin from the nanoparticles.

**Figure 5:**
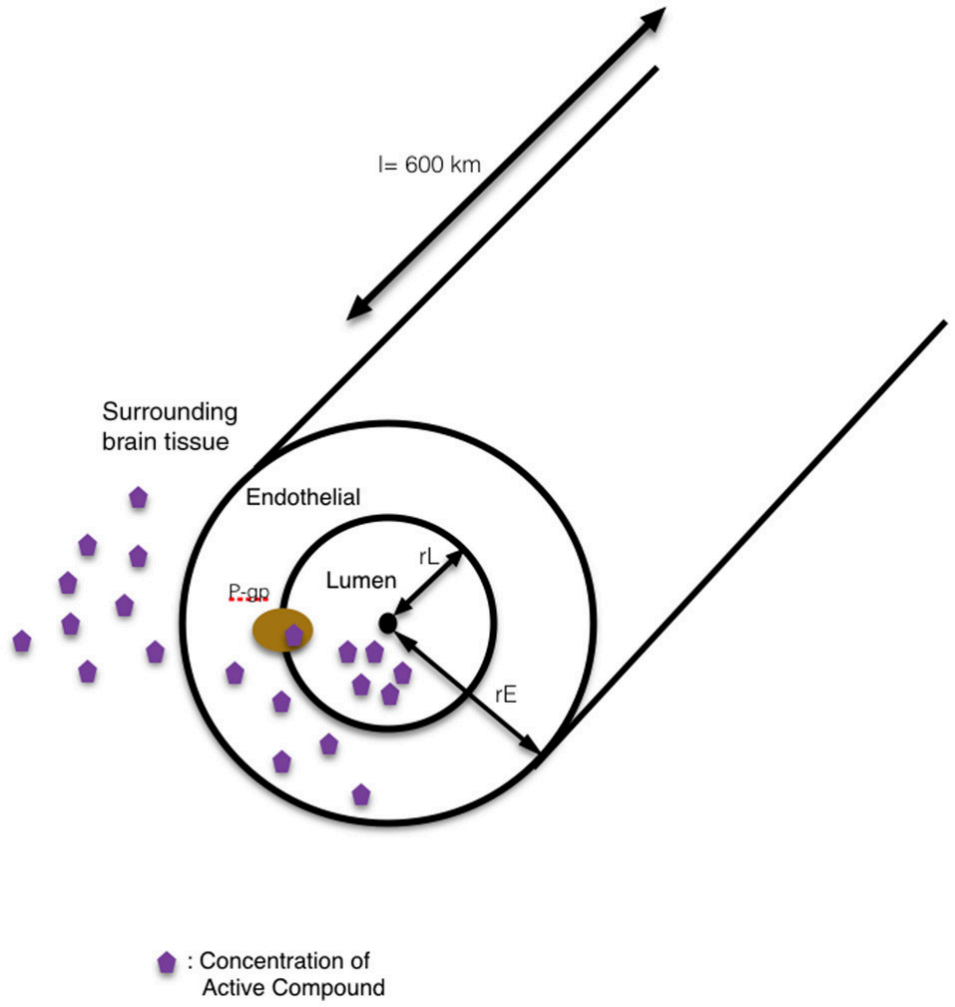
The schematic representation of our single-tube like capillary model. In this 1D model, the blood-brain barrier is simplified into a single blood vessel with a lumen inside and surrounding tissue outside. The wall of the vessel is represented as the endothelial side. The route for the transport of compound into the surrounding brain tissue by modulation of pgp-mediated efflux is shown in the schematic diagram.

Briefly, these models take the form of first order, chemical-kinetic, ordinary differential equation models. Convection-diffusion equations define the change in concentration of the delphinidin (1) and delphinidin-encapsulated nanoparticle (2) in the blood stream, and they are quantified in two compartments: in the lumen and in the endothelial cells of the brain capillaries. The right-hand side of the 1st equation includes three terms as diffusion, convection and creation or destruction of the quantity. Where ∇ is spatial gradient operator, ϑ is the velocity field that represents the convection or advection and R is the sources or sinks of the quantity C. The concentration in the lumen (L) and endothelial cell cytoplasm (E) of both free delphinidin (*C*_*L*,1_, *C*_*E*,1_) and delphinidin-encapsulated nanoparticles (*C*_*L*,2_, *C*_*E*,2_), respectively, were all considered. The concentration of the compound in the whole brain, CM, was considered constant. In the human brain, the total length of all capillaries is estimated to l = 600 km (Bandara et al., 2007). In our model, the geometry of the single-tube like capillary is shown in Figure 5, and its dimensions are defined by the luminal radius rL and the endothelial outer radius rE, the lumenal volume VL, the endothelial volume VE, the surface area between lumen and endothelia ALE, and the outer surface area of the capillary AEM. It is assumed that the transport by diffusion occurs across membranes only, and this is modeled between the vascular lumen and the cytoplasm of endothelial cells, and between endothelial cells and the surrounding brain tissue by the following equations:

(1) $$\begin{array}{l} \frac{dC}{dt}=\nabla .\left(D.\nabla C\right)-\nabla .\left(\vartheta C\right)+R \end{array}$$

(2) $$\begin{array}{l} J_{pgp}=\frac{J_{\max}.C_{E}}{K_{M}+C_{E}} \end{array}$$

(3) $$\begin{array}{l} \frac{dC_{L,1}}{dt}=\frac{1}{V_{L}}\left(A_{LE}.D_{LE,1}.\left(C_{E,1}-C_{L,1}\right)+\frac{J_{\max}.C_{E,1}}{K_{M}+C_{E,1}}l\right) \end{array}$$

(4) $$\begin{array}{l} \frac{dC_{L,2}}{dt}=\frac{1}{V_{L}}\left(A_{LE}.D_{LE,2}.\left(C_{E,2}-C_{L,2}\right)+\frac{J_{\max}.C_{E,2}}{K_{M}+C_{E,2}}l\right) \end{array}$$

(5) $$\begin{array}{l} \frac{dC_{E,1}}{dt}=\frac{1}{V_{E}}(A_{LE}.D_{LE,1}.\left(C_{L,1}-C_{E,1}\right)\\ \;+A_{EM}.D_{EM}.\left(C_{M,1}-C_{E,1}\right)-\frac{J_{\max}.C_{E,1}}{K_{M}+C_{E,1}}l) \end{array}$$

(6) $$\begin{array}{l} \frac{dC_{E,2}}{dt}=\frac{1}{V_{E}}(A_{LE}.D_{LE,2}.\left(C_{L,2}-C_{E,2}\right)\\ \;+A_{EM}.D_{EM}.\left(C_{M,2}-C_{E,2}\right)-\frac{J_{\max}.C_{E,2}}{K_{M}+C_{E,2}}l) \end{array}$$

Different diffusion coefficients for the transport across each membrane, *D*_*LE*1_, *D*_*LE*2_ and *D*_*EM*_, were defined as model parameters based on typical lipid composition in the membrane and speed of diffusion (Table 3).

**Table 3:** Model parameters and their meaning with known and estimated values where applicable.

**Parameter**	**Meaning**	**Value**	**Source**
D_LE free (D_LE_1)	Diffusion coefficient of free delphinidin between lumen and endothelial cytoplasm	0.5 μm/min	Reasonable prediction
D_LE encapsulated ( D_LE_2)	Diffusion coefficient of encapsulated delphinidin between lumen and endothelial	34.8 μm/min	Bandara et al., 2007
r_L	Luminal radius	1.2 μm	Estimated from histology
J_max	Maximum diffusion flux	2.42 pmol /min.dm	Bandara et al., 2007
D_EM	Diffusion coefficient between endothelial cytoplasm and outer membrane	1.89 μm/min	Bandara et al., 2007
r_E	Endothelial radius	3.9 μm	Estimated from histology
K_M	The Michaelis constant	1.50 μM	Bandara et al., 2007
C_M	The concentration in the surrounding brain tissue	1 μM	Bandara et al., 2007
k_1	The rate of release of delphinidin from endothelial cells' cytoplasm	0.01 /min	Dunn et al., 2006
k_2	The rate of p-gp uptake of delphinidin from endothelial cells' cytoplasm	0.035 /min	Dunn et al., 2006
k	Maximum transport rate due to p-gp pumping	0.21 μmol/min	Evans et al., 2009
t_start	Initial time	0 min	Nominal value
t_stop	Finish time	20 min	Nominal value

Binding and release, as well as active transport was modeled by linear chemical-kinetic relationships with the following equation:

(7) $$\begin{array}{l} \frac{dC_{u}^{*}}{dt}=-k_{1}\cdot C^{*}+k_{2}\cdot C_{u}^{*} \end{array}$$

where Cu^*^ = the concentration of delphinidin-encapsulated nanoparticle which are taken up by the efflux transporter p-gp, C^*^ = the concentration of released delphinidin-encapsulated nanoparticle, *k*_1_ = the rate of release of delphinidin from endothelial cells, *k*_2_ = the rate of p-gp uptake of delphinidin from endothelial cells. The model was implemented in the MATLAB. The full set of equations and MATLAB code are provided in the supplementary material.

**Figure 6:**
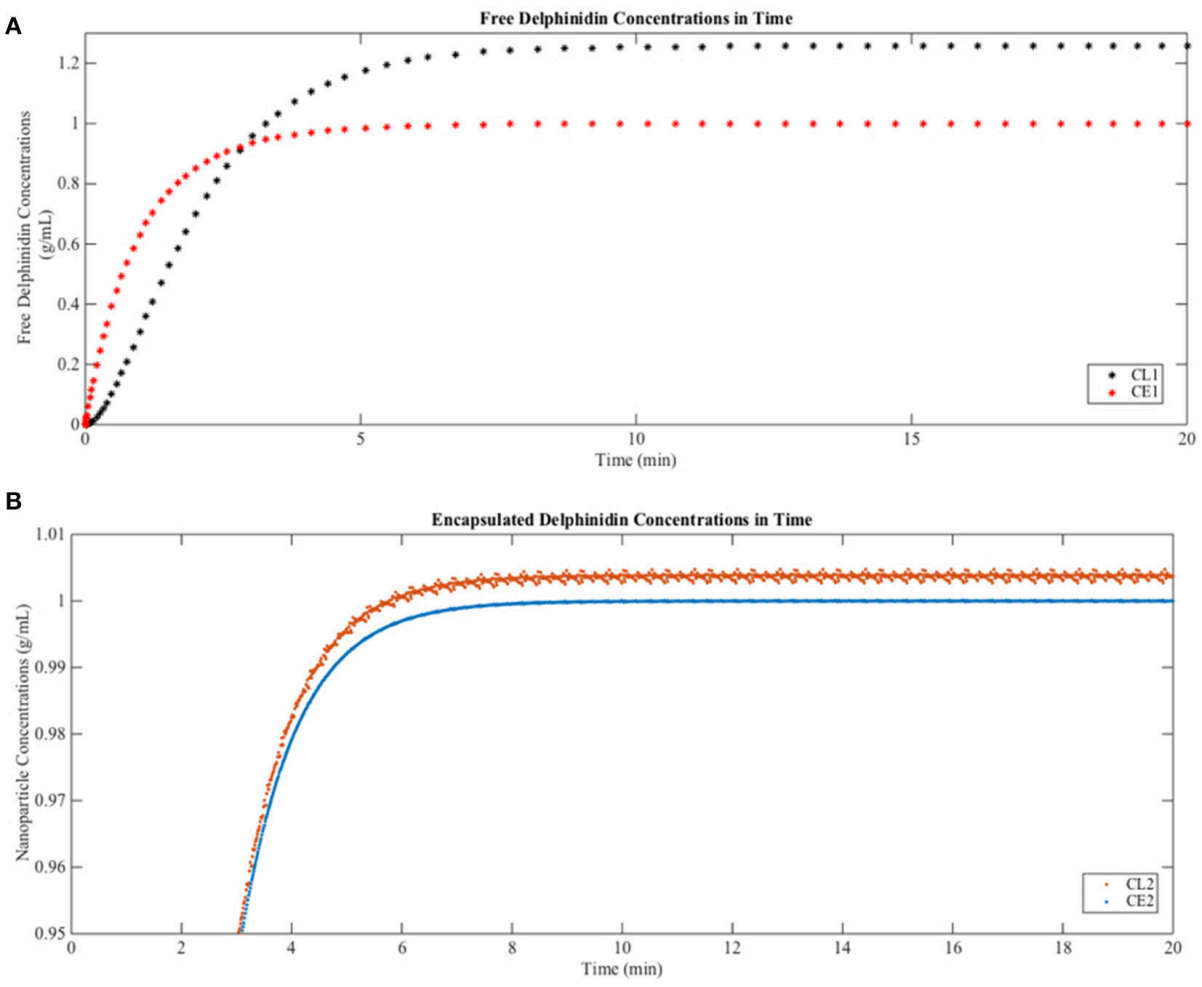
Predictions of free and encapsulated delphinidin delivery to brain tissue **(A)** Free delphinidin concentration vs. time graph where y-axis 0–1.3. **(B)** Encapsulated delphinidin concentration vs. time graph where y-axis 0.95–1.01. CL, concentration in the lumen; CE, concentration in the endothelial cells of the barrier.

When free delphinidin is encapsulated into a nanoparticle, the diffusion coefficient changes, and Figure 6 shows the predicted tissue concentration distributions from Equations (3–6) for the delphinidin free and encapsulated forms, using the parameters summarized in Table 3. To represent how encapsulation might cause effective diffusion into the brain, the free delphinidin diffusion coefficient was estimated for this system. As seen from the graph, our model predicts the distribution of delphinidin in its free and encapsulated forms. The maximum concentration of delphinidin in the brain is reached ~50.1 min following injection, a value obtained from solving Equation (7). Validation of the model is currently infeasible for humans, however future *in vivo* assays are merited to test these predictions and build on the quantitative theory.

We introduced this model to understand the transport of delphinidin in comparison to delphinidin-encapsulated nanoparticles across the BBB quantitatively, which is controlled by diffusion, active transport, and p-gp binding. It is important to emphasize that all models are idealized representations of reality, and the models described here are significant simplifications of complex biology and geometry. Hereby, adjusting nanoparticle shape, size, and other properties could result in avoiding p-gp blocking at the BBB and successfully passing through the membrane for the therapy of glioblastoma with delphinidin. While building from a similar foundation, future models of nanomedicine delivery may consider many of the other microenvironmental and cellular factors involved in effective brain tumor-targeted drug delivery (Liu and Lu, 2012). Along with the BBB, other major membranes which impose obstacles to brain tumor treatment include the blood-cerebrospinal fluid barrier (Béduneau et al., 2007) and blood-tumor barrier (Van Tellingen et al., 2015). Additionally, an enhanced permeability and retention (EPR) effect (Torchilin, 2011) could be incorporated in subsequent models to optimize predictions for the delivery of new therapeutic agents. *In vitro* experiments to estimate kinetic rates can be used to validate and/or improve parameters of the models and include more complex terms like possible interactions of the nanoparticles with local tissue (adsorption/ desorption), nanoparticle swelling and erosion.

## Conclusion

This review summarizes examples of current advances in the glioblastoma therapy to increase our understanding of molecular mechanisms underlying glioma progression and explore the potential of new nanomedicines. We have highlighted the use of two systems biology tools, network signaling diagrams, and mathematical models, in distinguishing differential glioma cell signaling and in predicting delivery of delphinidin to brain tissue. In this regard, quantitative tools open up a new avenue for glioblastoma research, and provide an essential method to explore the potential of nanomedicine in brain cancer treatment.

## Author contributions

EO-K and AQ: Drafted the manuscript; EO-K: Design and made the figures and tables. All authors read and revised the manuscript and approved its content.

### Conflict of interest statement

The authors declare that the research was conducted in the absence of any commercial or financial relationships that could be construed as a potential conflict of interest.

## Footnotes

^1^CancerGenomics at cBio - Glioblastoma (TCGA) [WWW Document], n.d. URL Available online at: http://cbio.mskcc.org/research/cancergenomics/gbm/pathways/index.html (Accessed February 28, 2018).
