# Supplementary material for: Advances in Glioblastoma Multiforme Treatment: New Models for Nanoparticle Therapy
Source: Front Physiol. 2018 Mar 19;9:170. doi: 10.3389/fphys.2018.00170 (PMC5868458; doi:10.3389/fphys.2018.00170)
Supplement: Supplementary file 1 [file DataSheet1.DOCX]

**Supplementary materials for advances in glioblastoma multiforme treatment: new models for nanoparticle therapy**

**Additional Equations**

$$\frac{{dC}_{L,1}}{dt}=\frac{2.D_{LE,1}}{r_{L}}\left( C_{E,1}-C_{L,1} \right)+\frac{C_{E,1}}{K_{M}+C_{E,1}}.\frac{J_{max}}{\pi r_{L}^{2}}$$

$$\frac{{dC}_{L,2}}{dt}=\frac{2.D_{LE,2}}{r_{L}}\left( C_{E,2}-C_{L,2} \right)+\frac{C_{E,2}}{K_{M}+C_{E,2}}.\frac{J_{max}}{\pi r_{L}^{2}}$$

$$\frac{{dC}_{E,1}}{dt}=\frac{2{.r}_{L}.D_{LE,1}}{r_{E}^{2}-r_{L}^{2}}\left( C_{L,1}-C_{E,1} \right)+\frac{2.r_{E}.D_{EM,1}}{r_{E}^{2}-r_{L}^{2}}.\left( C_{M,1}-C_{E,1} \right)-\frac{C_{E,1}}{K_{M}+C_{E,1}}.\frac{J_{max}}{\pi\left( r_{E}^{2}-r_{L}^{2} \right)}$$

$$\frac{{dC}_{E,2}}{dt}=\frac{2{.r}_{L}.D_{LE,2}}{r_{E}^{2}-r_{L}^{2}}\left( C_{L,2}-C_{E,2} \right)+\frac{2.r_{E}.D_{EM,2}}{r_{E}^{2}-r_{L}^{2}}.\left( C_{M,2}-C_{E,2} \right)-\frac{C_{E,2}}{K_{M}+C_{E,2}}.\frac{J_{max}}{\pi\left( r_{E}^{2}-r_{L}^{2} \right)}$$

**MATLAB Codes**

**First Model**

%Diffusion, transport and release of active compound both free and

%nanoparticle encapsulated form.

%Delphinidin (1) and delphinidin-encapsulated nanoparticle(2) would be quantified

%in two compartments: in the lumen and in the endothelial cells of the brain capillaries.

% Solving simultaneous differential equations

%For delphinidin free form:

function dCAdt_vector=ode_function_a (t, CA_vector)

CL1=CA_vector(1); %CL1: The free delphinidin concentration in the Lumen

CE1=CA_vector(2); %CE1: The free delphinidin concentration in the Endothelial

%Defining estimated parameters:

DLE=0.5;

rL=1.2;

Jmax=2.42;

DEM=1.89;

rE=3.9;

KM=1.50;

CM=1;

r2=rE^2-rL^2;

dCL1dt=(2*DLE/rL*(CE1-CL1))+ (CE1*Jmax/(KM+CE1)/pi/rL^2); %DLE: diffusion between Lumen and Endothelial

dCE1dt=(2*rL*DLE/r2*(CL1-CE1))+ (2*rE*DEM*(CM-CE1)/r2)-(CE1*Jmax/(KM+CE1)/pi/r2); %DEM: Diffusion between Endothelial and surrounding Medium

dCAdt_vector = [dCL1dt; dCE1dt];

end

%Save ode_function_a as M-file;

function solve_coupled_aodes

tstart=0;

tstop=20;

initial_CA_vector =[0;0];

[times, sols]= ode45(@ode_function_a,[tstart, tstop], initial_CA_vector);

plot(times, sols(:,1),'k*');

hold on

plot(times, sols(:,2),'r*');

legend ('CL1', 'CE1');

title ('Free Delphinidin Concentrations in Time');

xlabel('Time (min)');

ylabel('Free Delphinidin Concentrations (g/mL)');

axis([0,20,0.8,1.26])

end

%Save solve_coupled_aodes as M-file

%For solution; write solve_coupled_aodes in command window and press enter

%Diffusion, transport and release of active compound both free and

%nanoparticle encapsulated form.

%Delphinidin (1) and delphinidin-encapsulated nanoparticle(2) would be quantified

%in two compartments: in the lumen and in the endothelial cells of the brain capillaries.

% Solving simultaneous differential equations

%For delphinidin nanoparticle encapsulated form:

function dCNdt_vector=ode_function_n(t, CN_vector)

CL2=CN_vector(1); %CL2: The nanoparticle encapsulated delphinidin concentration in the Lumen

CE2=CN_vector(2); %CE2: The nanoparticle encapsulated delphinidin concentration in the Endothelial

%Defining estimated parameters:

DLE=34.8;

rL=1.2;

Jmax=2.42;

DEM=1.89;

rE=3.9;

KM=1.50;

CM=1;

r2=rE^2-rL^2;

dCL2dt=(2*DLE/rL*(CE2-CL2))+ (CE2*Jmax/(KM+CE2)/pi/rL^2); %DLE: diffusion between Lumen and Endothelial

dCE2dt=(2*rL*DLE/r2*(CL2-CE2))+ (2*rE*DEM*(CM-CE2)/r2)-(CE2*Jmax/(KM+CE2)/pi/r2); %DEM: Diffusion between Endothelial and surrounding Medium

dCNdt_vector = [dCL2dt; dCE2dt];

end

%Save ode_function_n as M-file;

function solve_coupled_nodes

tstart=0;

tstop=20;

initial_CN_vector =[0;0];

[times, sols]= ode45(@ode_function_n,[tstart, tstop], initial_CN_vector);

figure

plot(times, sols(:,1),'g*');

hold on

plot(times, sols(:,2),'b*');

legend ('CL2', 'CE2');

title ('Encapsulated Delphinidin Concentrations in Time);

xlabel('Time (min)');

ylabel('Nanoparticle Concentrations (g/mL)');

axis([0,20,0.9,1.01]);

end

%Save solve_coupled_nodes as M-file

%For solution; write solve_coupled_nodes in command window and press enter

**Second Model**

%The analytical solutions to the differential equations with the MATLAB

%command dsolve:

%A=amount of drug in the absorption site (lumen); B=amount of drug released

%in the brain; E= amount of drug uptaked by pgp (eliminated);

%k1= absorption rate; k2= uptake rate; k= max transport rate due to pgp

%pumping

[A, B, E]= dsolve ('DA=-k1*A','DB=k1*A-k2*B', 'DE=k2*B', 'A(0)=A0', 'B(0)=0', 'E(0)=0');

A=simplify(A); %A=A0*exp(-k1*t)

B=simplify (B); %(A0*k1*exp(-t*(k1 + k2))*(exp(k1*t) - exp(k2*t)))/(k1 - k2)

E=simplify (E); %exp(-k1*t)*exp(-k2*t)*(A0*exp(k1*t)*exp(k2*t) - (A0*k1*exp(k1*t))/(k1 - k2) + (A0*k2*exp(k2*t))/(k1 - k2))

simplify (A+B+E); %A(t)+B(t)+E(t)= A0+B0+E0 ; (remember B0 and E0 are equal zero, thats why ans = A0

%The value of tmax is obtained by taking derivative of B(t), equating it to

%zero, and solving for t, using the values k1=0.01 and k2=0.035; and

%k=0.21

dB = diff(B); % dB =(A0*k1*exp(-t*(k1 + k2))*(k1*exp(k1*t) - k2*exp(k2*t)))/(k1 - k2) - (A0*k1*exp(-t*(k1 + k2))*(exp(k1*t) - exp(k2*t))*(k1 + k2))/(k1 - k2)

tmax = solve(dB,'t'); % tmax=(log(k1/k2) + pi*k*2*i)/(k1 - k2)

k1=0.01;k2=0.035;k=0.21;

eval(tmax)

%This predicts that the max conc. of the drug in the brain is reached

%approximately (tmax) minutes later after injection.
